# Recipients Affect Prosocial and Altruistic Choices in Jackdaws, *Corvus monedula*

**DOI:** 10.1371/journal.pone.0034922

**Published:** 2012-04-12

**Authors:** Christine Schwab, Ruth Swoboda, Kurt Kotrschal, Thomas Bugnyar

**Affiliations:** 1 KLI for Evolution and Cognition Research, Altenberg, Austria; 2 Konrad Lorenz Research Station for Ethology, Gruenau, Austria; 3 Department of Behavioural Biology, University of Vienna, Vienna, Austria; 4 Department of Cognitive Biology, University of Vienna, Vienna, Austria; University of Lethbridge, Canada

## Abstract

Other-regarding preferences are a critical feature of human cooperation but to what extent non-human animals exhibit these preferences is a matter of intense discussion. We tested whether jackdaws show prosocial behaviour (providing benefits to others at no cost to themselves) and altruism (providing benefits to others while incurring costs) with both sibling and non-sibling recipients. In the prosocial condition, a box was baited on both the actor's and the recipient's side (1/1 option), whereas another box provided food only for the actor (1/0 option). In the altruistic condition, the boxes contained food for either the actor (1/0 option) or the recipient (0/1 option). The proportion of selfish (1/0 option) and cooperative (1/1 and 0/1 option, respectively) actors' choices was significantly affected by the recipients' behaviour. If recipients approached the boxes first and positioned themselves next to the box baited on their side, trying to access the food reward (recipient-first trials), actors were significantly more cooperative than when the actors approached the boxes first and made their choice prior to the recipients' arrival (actor-first trials). Further, in recipient-first trials actors were more cooperative towards recipients of the opposite sex, an effect that was even more pronounced in the altruistic condition. Hence, at no cost to the actors, all recipients could significantly influence the actors' behaviour, whereas at high costs this could be achieved even more so by recipients of different sex. Local/stimulus enhancement is discussed as the most likely cognitive mechanism to account for these effects.

## Introduction

Human cooperation is characterized by a high degree of prosocial and altruistic behaviour [1], [2]. Research on the evolutionary origin of cooperation comprises an extensive amount of studies on diverse taxa known to exhibit cooperative behaviours (see reviews: [3]–[6]. However, studies that investigate other-regarding preferences in choice tasks, i.e. a concern for the welfare of others [7], so far have been conducted only on non-human primates and follow two main hypotheses. The first hypothesis states that prosocial behaviour (when an actor provides benefits to others at no cost to itself) and even altruistic behaviour (when an actor provides benefits to others by enduring costs and without receiving direct benefits) have their evolutionary roots in the primate line and may be shared traits of humans and their closest living relatives, i.e. chimpanzees, *Pan troglodytes* [8]–[13]. However, although chimpanzees in the wild exhibit a range of cooperative behaviours, such as coalition formation, food sharing, cooperative hunting, or communal defense of territories [14]–[17], results from laboratory experiments on other-regard remain ambiguous. In a food context, chimpanzees in most studies did not exhibit any other-regarding preferences [9]–[11], [18], but they did show prosocial behaviour in a recent study [19]. In a non-food context, however they often assist familiar as well as unfamiliar humans to access objects that are out of reach [13], [20]. They may also transfer tools needed to access food to conspecifics [21].

The second hypothesis assumes that cooperative breeders are motivated and psychologically predisposed to act prosocially [22]. Testing cooperatively breeding challithrichid monkeys in other-regarding tasks, however, yielded ambiguous results. While common marmosets, *Callithrix jacchus*, fitted the hypothesis and altruistically provided food to kin and non-kin partners [7], cottontop tamarins, *Saguinus oedipus*, did not [23], or their altruistic food provisions were to a large extent influenced by reciprocal exchange [24], [25]. Moreover, non-cooperatively breeding primates were also found to behave prosocially under certain conditions. Brown capuchin monkeys, *Cebus apella*, showed prosocial food choices towards familiar kin and non-kin but not towards unfamiliar individuals [12], and long-tailed macaques, *Macaca fascicularis*, directed prosocial behaviour mainly towards kin [26] and dominant individuals [27].

What has been fairly neglected in these studies on other-regard is the role of the recipients. Melis and colleagues (2011) recently proposed a third hypothesis (signalling hypothesis): recipients should signal their goals or requests for help to their conspecific partner because the acting subjects might have limitations in their ability to infer the recipients' goals and needs in the absence of overt actions or requests. In chimpanzees they found a significant positive effect of the recipient's behaviour on eliciting altruistic behaviour in a conspecific actor in a food and a non-food context [8], which confirms some studies [19]–[21] but contradicts others [10]. In the few studies on primates other than chimpanzees in which the recipients' behaviour was analyzed, results either showed no influence on the actors' behaviour [7] or even a reduction in altruistic choices [23].

Corvids are large-brained birds [28] with primate-like cognitive abilities [29], [30], particularly in the social domain [31]. Importantly, they exhibit a variety of cooperative behaviours: ravens, *Corvus corax*, recruit conspecifics to foraging sites [32]–[34] and some populations of carrion crows, *Corvus corone*, are cooperative breeders [35]. Further, jackdaws and other corvid species show social support in agonistic interactions [36], [37], prolonged parental care of offspring [38], communal mobbing [39], and various forms of food-sharing [40]–[42]. In jackdaws, *Corvus monedula*, food sharing includes a high percentage of actor-initiated behaviours [40], making this species particularly interesting for investigating other-regarding preferences. Furthermore, recipient-initiated sharing has been demonstrated in jackdaws in an experimental set-up and is functionally explained by harassment avoidance [41]. Accordingly, we aimed at testing aspects of the signalling hypothesis, i.e. whether other-regarding preferences, expressed as prosocial and altruistic choices, rely on the behaviour of the recipient.

**Figure 1:**
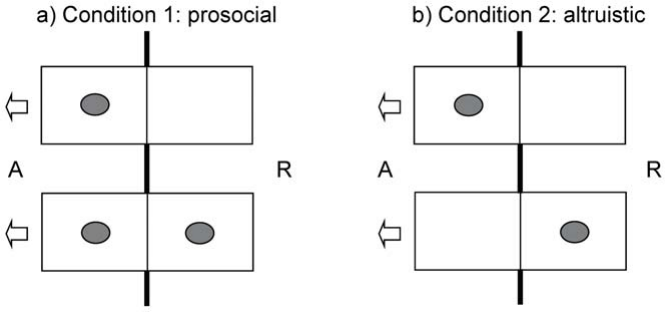
Sketch of baited boxes in both test conditions. Grey items display the food reward, A indicates the actor and R indicates the recipient side. Arrows describe the only direction in which the boxes could be opened: only by the actor and to the actor's side. Bold lines above, between, and below the drafted boxes illustrate the dividing wire mesh between test compartments while intermediate thin lines illustrate the transparent partitions within boxes. Distance between the boxes is not to scale. a) represents condition 1, the prosocial condition, with the 1/0 option on top and the 1/1 option below. b) represents condition 2, the altruistic condition, with the 1/0 option on top and the 0/1 option below.

We subjected seven birds (four females, three males) to a prosocial and an altruistic test condition in which the respective actor individual could choose to open one of two boxes that were baited with preferred and identical food items (dry cat food). Boxes were divided into two compartments, one of which could be accessed by the actor, the other by the recipient (Fig. 1). In the prosocial condition, one of the boxes was baited with one food reward each on both the actor's and the recipient's side (hereafter referred to as the ‘1/1 option’), whereas the second box provided food only for the actor (1/0 option, Fig. 1a). If jackdaws show prosocial behaviour, actors should choose the 1/1 option, which reflects the cooperative choice more often than the 1/0 option, which reflects the selfish choice. In the altruistic condition, a box was baited either on the actor's (1/0 option) or on the recipient's side (0/1 option, Fig. 1b). If jackdaws show altruistic behaviour, in this condition actors should choose the 0/1 option, which reflects the cooperative choice, more often than the 1/0 option, which again reflects the selfish choice. In both conditions the actors were tested with a sibling and a non-sibling recipient but the roles of actors and recipients were never reversed to exclude a potential influence of short-term reciprocity within the experimental context.

Boxes were placed onto a wooden platform that extended into both experimental compartments, each of which accommodated one bird. Boxes were therefore accessible by both birds and allowed either the actor or the recipient to approach them first. In actor-first trials the actor approached the boxes first and made its choice prior to the recipient landing on the platform. In recipient-first trials the recipient approached the boxes first and, although the boxes could only be opened from the actor's side, positioned itself next to the box baited on its side and/or tried to get access to the food reward by pecking/stepping onto the box. Therefore, when the actor later arrived and made its choice, the recipient was already present at its baited box.

Before proceeding to the test conditions actor individuals received two training phases (see methods for details) to ensure that they were able to correctly manipulate the boxes and that they had learned to make only one choice per trial. Data were analyzed by conducting a generalized linear mixed model (GLMM) and Wilcoxon signed-ranks tests. See methods for details.

## Results

Recipient-first trials constituted 37% of the trials with siblings and 34% of the trials with non-siblings in the prosocial condition. In the altruistic condition they comprised 19% of the trials with siblings and 27% of the trials with non-siblings.

**Figure 2:**
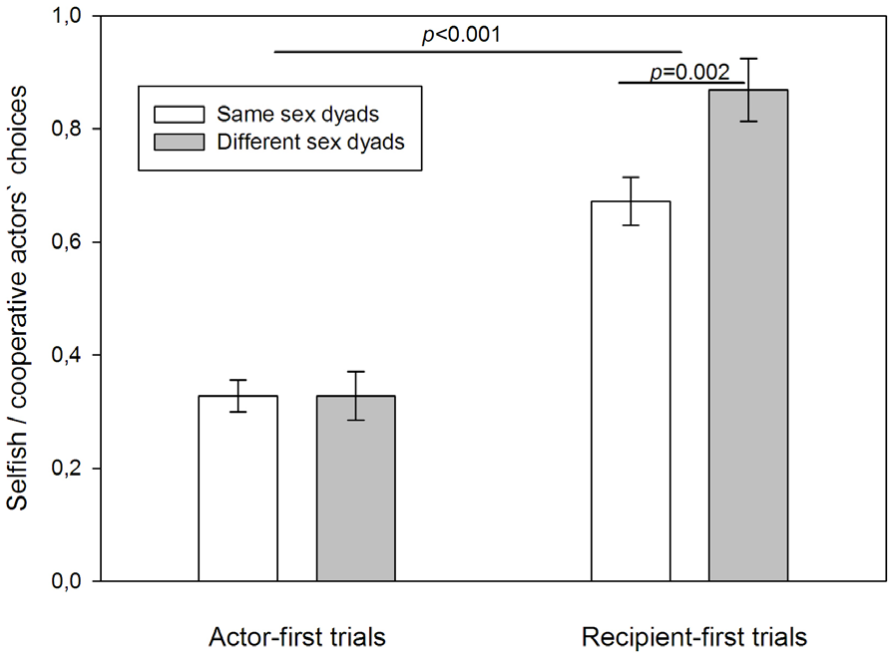
Actors' choices by first approach and sex relation of dyads. In actor-first trials actors approached the boxes first and made their choice prior to the arrival of the recipient. In recipient-first trials recipients approached the boxes first and the later arriving actors made their choice with the recipients present at their baited box. White bars depict same sex dyads, grey bars depict different sex dyads. The Y-axis displays the proportion of selfish versus cooperative choices and ranges from 0 to 1. 0 represents solely selfish and 1 represents solely cooperative choices. Bars constitute mean values ± SE. P-values derive from the final model and pairwise tests.

The proportion of actors' selfish (1/0 option) and cooperative (1/1 and 0/1 option, respectively) choices was most strongly influenced by first approach (F_1, 548_ = 42.702, *p*<0.001, Tab. 1): actors were significantly more cooperative in recipient-first (mean ±SE: 0.804±0.048) than in actor-first trials (mean ± SE: 0.327±0.026, Fig. 2).

**Table 1:** Fixed terms of the generalized linear mixed model (GLMM) to determine possible influences on actors' choices (*N* = 560 trials and 7 individuals).

Fixed term	Full fixed model			Final model		
	F	df1,df2	p	F	df1,df2	p
First approach	41.711	1, 541	<0.001	**42.702**	**1, 548**	**<0.001**
Condition	0.38	1, 541	0.538	0.323	1, 548	0.57
Kinship	0.049	1, 541	0.824	*0.427*	*1, 547*	*0.514*
Sex relation	4.74	1, 541	0.03	**4.609**	**1, 548**	**0.032**
Sex of actor	0.016	1, 541	0.899	0.201	1, 548	0.654
First approach*condition	0.915	1, 541	0.339	0.836	1, 548	0.361
First approach*kinship	0.438	1, 541	0.508	*0.291*	*2, 546*	*0.748*
First approach*sex relation	4.995	1, 541	0.026	**5.009**	**1, 548**	**0.026**
First approach*sex of actor	0.01	1, 541	0.919	0.01	1, 548	0.921
Condition*kinship	0.884	1, 541	0.347	*0.213*	*2, 546*	*0.808*
Condition*sex relation	2.702	1, 541	0.101	2.228	1, 548	0.136
Condition*sex of actor	1.437	1, 541	0.231	1.809	1, 548	0.179
Kinship*sex relation	0.081	1, 541	0.777	*0.259*	*2, 546*	*0.772*
Kinship*sex of actor	0.191	1, 541	0.662	*0.317*	*2, 546*	*0.729*
Sex relation*sex of actor	0.258	1, 541	0.612	*0.625*	*1, 547*	*0.43*
First approach*condition*kinship	2.51	1, 541	0.114	*0.826*	*4, 544*	*0.509*
First approach*condition*sex relation	3.518	1, 541	0.061	**3.154**	**1, 548**	**0.076**
First approach*condition*sex of actor	7.317	1, 541	0.007	**8.169**	**1, 548**	**0.004**

The binomial response variable was selfish/cooperative actor's choice in the prosocial and the altruistic condition. Actor identity was included as a random term in all models. For the full model, results of all tested fixed terms are given. For the final model, results of terms that remained in the final model are given straight with significant effects (p<0.05) and non-significant tendencies (0.05<p<0.1) in bold. Results of excluded terms when individually re-entered into the final model are given in italics.

Analyzing all actor-first trials together showed that actors chose the selfish option significantly more often than the cooperative one (Wilcoxon signed-ranks test: N = 7, *Z* = −2.366, *p* = 0.018). On the contrary, in recipient-first trials actors showed a nearly significant tendency to choose the cooperative option more often than the selfish one (Wilcoxon signed-ranks test: N = 7, *Z* = −1.859, *p* = 0.063). In the control condition that resembled the prosocial condition without a recipient present, actors did not show a significant preference for either the 1/0 or the 1/1 option (Wilcoxon signed-ranks test: N = 5, *Z* = −1.633, *p* = 102).

Our analyses further showed that the interaction of first approach and the sex relation of dyads (same sex vs. different sex dyads) significantly affected actors' choices (F_1, 548_ = 5.009, *p* = 0.026, Tab. 1): in recipient-first trials actors were significantly more cooperative in different sex (mean±SE: 0.9±0.054) than in same sex dyads (mean±SE: 0.653±0.05, F_1, 548_ = 9.957, *p* = 0.002, Fig. 2) but there was no significant difference in actor-first trials (same sex dyads mean±SE: 0.33±0.032, different sex dyads mean±SE: 0.323±0.044, F_1, 548_ = 0.016, *p* = 0.901, Fig. 2).

**Figure 3:**
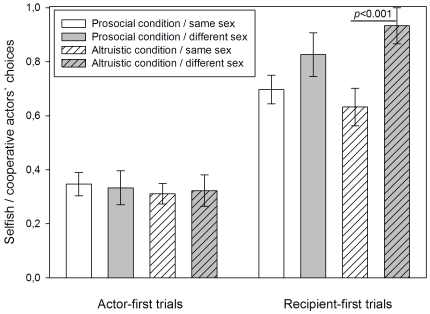
Actors' choices by first approach, condition, and sex relation of dyads. In actor-first trials actors approached the boxes first and made their choice prior to the arrival of the recipient. In recipient-first trials recipients approached the boxes first and the later arriving actors made their choice with the recipients present at their baited box. White bars depict same sex dyads, grey bars depict different sex dyads. Plain bars illustrate the prosocial condition, striped bars illustrate the altruistic condition. The Y-axis displays the proportion of selfish versus cooperative choices and ranges from 0 to 1. 0 represents solely selfish and 1 represents solely cooperative choices. Bars constitute mean values ± SE. P-values derive from pairwise tests of the final model.

Condition per se did not have a significant effect on actors' choices, neither as main term nor in first order interactions (Tab. 1) but its inclusion in the second order interaction of first approach*condition*sex relation of dyads revealed a non-significant tendency to affect actors' choices (F_1, 548_ = 3.154, *p* = 0.076, Tab. 1). Pairwise contrasts showed that this effect was highly significant only in the altruistic condition of recipient-first trials (same sex dyads mean±SE: 0.573±0.085, different sex dyads mean±SE: 0.953±0.047, F_1, 548_ = 13.345, *p*<0.001, Fig. 3) in which actors were again more cooperative towards different sex than towards same sex recipients. In the prosocial condition of recipient-first trials, however, actors' choices did not differ between same sex dyads (mean±SE: 0.726±0.054) and different sex dyads (mean±SE: 0.797±0.091, F_1, 548_ = 0.445, *p* = 0.505, Fig. 3). They also did not significantly differ in actor-first trials, either in the prosocial (same sex dyads mean±SE: 0.332±0.049, different sex dyads mean±SE: 0.347±0.067, F_1, 548_ = 0.03, *p* = 0.863) or in the altruistic condition (same sex dyads mean±SE: 0.329±0.04, different sex dyads mean±SE: 0.3±0.058, F_1, 548_ = 0.156, *p* = 0.693, Fig. 3).

**Figure 4:**
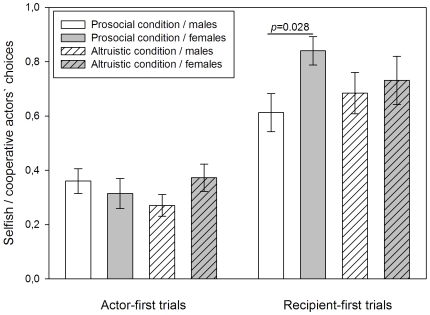
Actors' choices by first approach, condition, and sex of actors. In actor-first trials actors approached the boxes first and made their choice prior to the arrival of the recipient. In recipient-first trials recipients approached the boxes first and the later arriving actors made their choice with the recipients present at their baited box. White bars depict male actors, grey bars depict female actors. Plain bars illustrate the prosocial condition, striped bars illustrate the altruistic condition. The Y-axis displays the proportion of selfish versus cooperative choices and ranges from 0 to 1. 0 represents solely selfish and 1 represents solely cooperative choices. Bars constitute mean values ± SE. P-values derive from pairwise tests of the final model.

Furthermore, the second order interaction of first approach*condition*sex of actor (female vs. male actors) also yielded a significant effect on actors' choices (F_1, 548_ = 8.169, *p* = 0.004, Tab. 1) but again only in recipient-first trials. Here, pairwise contrasts showed that female actors made more prosocial choices (mean±SE: 0.85±0.052) than male actors (mean±SE: 0.647±0.088, F_1, 548_ = 4.863, *p* = 0.028, Fig. 4) whereas there was no significant difference between female and male actors' choices in the altruistic condition (females mean±SE: 0.765±0.105, males mean±SE: 0.894±0.064, F_1, 548_ = 1.704, *p* = 0.192, Fig. 4). In actor-first trials, however, there were no significant differences between female and male actors' choices in either condition (prosocial condition, females mean±SE: 0.313±0.056, males mean±SE: 0.367±0.057, F_1, 548_ = 0.4, *p* = 0.527; altruistic condition, females mean±SE: 0.372±0.05, males mean±SE: 0.262±0.045, F_1, 548_ = 2.693, *p* = 0.101, Fig. 4).

## Discussion

Taken together, our results concerning actor-first trials indicate that jackdaws did not actively show other-regarding preferences but chose the selfish option more often than the cooperative option. However, all recipients could significantly influence the actors' choices to their own advantage and particularly when they were of different sex, an effect that was even more pronounced in the altruistic condition, when costs for the actors were high (recipient-first trials).

That jackdaws did not exhibit any other-regarding preferences per se is in line with results from some studies on chimpanzees [9], [10]. It is difficult to interpret, however, why the birds' choices in actor-first trials were actually asocial. A possible explanation may be a carry-over effect from the second step of the training phase in which the actors learned that they were allowed to make a single choice (for details see methods) and they could have developed a preference for the 1/0 option as the rewarding and safe option. This explanation seems unlikely, however, if we consider the results of the control condition. Those trials were intermixed with the other-regard trials and tested whether the birds would show a preference for the box containing the greater amount of food (2 food items in the 1/1 option compared to 1 food item in the 1/0 option). Actors were given the same choice options as in the prosocial condition but were tested singly, in the absence of a recipient. They did not show a significant preference for either of the two boxes, which confirms that the birds were not simply going for the side with more food and also renders the explanation of a learned preference for the 1/0 option unlikely.

Focusing on the recipient-first trials, our results are in accordance with a similarly significant effect of recipients' behaviour on actors' choices in chimpanzees [8], [19]. Hence, our results may support the signalling hypothesis as a proximate mechanism eliciting other-regarding preferences that requires recipients to signal their goals or requests for help to their conspecific partner [8]. A signal is an actively given vehicle for transporting information that can be manipulated by the sender and serves particular functions, whereas cues contain information but are not actively given and are not necessarily of advantage to the sender [43], [44]. Although Melis and colleagues consider that the recipient's behaviour may provide a cue for the actor to ‘do something’, they interpret their findings as intention-reading and goal-understanding from the actor's view [8].

In our study, jackdaw recipients showed their interest in the food by positioning themselves at the baited box and/or trying to get access to the reward by pecking or stepping onto the box. However, they did not emit any begging calls, nor did they show any begging-related behavioural displays [45], providing no evidence for active signalling or harassment towards the actor. Although the recipients' behaviours likely represent cues about their motivation to get the food, we cannot conclude on the basis of the current findings whether or not those cues were interpreted as requests by the actor. However, what the recipients' behaviours clearly did is drawing the actors' attention to a particular box, thereby resembling local/stimulus enhancement and increasing the likelihood of opening that box. Following Zentall's definition (1996), the term stimulus enhancement is used when the activity of the demonstrator draws the attention of the observer to a particular *object*, whereas with local enhancement, the attention is drawn to a particular location [46]. Enhancement is one of the main proximate mechanisms facilitating social learning in animals [47], [48] and has been described as a major component in the social foraging patterns of corvids in general [49]–[51] and jackdaws in particular [37], [39], [52]. For our results, we therefore favour a lower-level explanation [53] and suggest local or stimulus enhancement as the most plausible mechanism.

Interestingly, females actors were more affected by the recipients' cues in the prosocial condition than in the altruistic condition (Fig. 4) but only in the latter did both sexes distinguish between recipients of same sex and of different sex, being more cooperative with different sex recipients (Fig. 2), especially in the altruistic condition (Fig. 3). These findings suggest that jackdaws did pay attention to the identity of the recipient and their own costs. Hence, having their attention directed to a particular box by a recipient does not automatically lead to an action at that location but goes along with a decision process in which the birds take into account the other's identity and the costs associated with their choices in the different conditions. Enhancement seems to be a weaker mechanism to induce cooperative behaviour towards same sex individuals, in particular at high cost to the actors. Functionally, this makes sense because food sharing in jackdaws has been suggested to facilitate the formation of social bonds [40]. Since the birds in this experiment were sub-adults but not yet paired, they may have been especially attracted to different sex recipients as potential future pair partners. This might explain why there were no significant effects on actors' choices by kinship (Tab. 1). Possibly, at this age strong kinship relations found in juvenile birds [52] have already dissolved, giving way to pair bond formation. The upcoming pair bonding period may have also increased competition between same-sex individuals which adds costs to cooperative acts and may additionally explain the result of less cooperative choices towards individuals of the same sex.

In sum, this first study on other-regard in birds corroborates results from several experiments on non-human primates insofar that jackdaws did not show other-regarding preferences per se. However, they did respond to enhancement cues provided by recipients and increased their prosocial choices with either type of recipient and altruistic choices even more with recipients of different sex. The set-up used in this study aimed to control for reciprocity. Further studies are needed to investigate whether the same mechanisms would work in a more naturalistic setting, which allows individuals to interact freely and repeatedly.

## Methods

### Ethics statement

This study complied with Austrian and local government guidelines and permission was received from the Konrad Lorenz Forschungstelle and the ethics committee of BH Gmunden (Permit Number Sich71-1309) specifically approved testing the jackdaws for this study.

### Subjects and Housing

Subjects were 12 jackdaws (*Corvus monedula*, 5 females, 7 males) that were housed together with six further conspecifics in one social group in an outdoor aviary at the Konrad Lorenz Research Station (KLF), Gruenau, Austria. All individuals were handraised in spring 2007 at the KLF under standardized conditions with sibling groups being raised together but in separate nests, i.e. cardboard boxes, to allow for familiarization with and later recognition of kin. The aviary consisted of one outdoor compartment (60 m^2^, maximum height of 4 m) which was equipped with wooden perches, breeding boxes, rocks and natural vegetation and an outdoor experimental area (30 m^2^, 2.5 m high) which was roofed and equipped with wooden perches and could be divided into smaller compartments via wooden doors. When not being tested in behavioural experiments, birds could move around freely in all areas. They had *ad libitum* access to water and were fed three times a day. Their diet consisted of dry insects, minced beef, various kinds of fruits, vegetables, grain, eggs, bread, and milk products.

### Composition of Dyads

Each of the seven test subjects (actors; 4 females, 3 males) was composed into a dyad with a conspecific (recipient) which was chosen out of a set of 5 birds with regards to meeting certain requirements due to the experimental set up. Actors and recipients were of same age and recipients were either the actor's sibling (kin) or non-sibling (non-kin) and of the same or different sex. Each subject was tested in two dyads (with a sibling and with a non-sibling). The specific actor-recipient dyads were maintained over the entire experiment and actor-recipient roles were not reversed to exclude the possibility of short-term reciprocation within the context of the experiment. Each set of sibling and non-sibling dyads consisted of 3 male-male, 2 female-female, and 2 male-female dyads.

### Experimental procedure and testing apparatus

Individuals from a dyad were placed in two adjacent compartments (actor's compartment, recipient's compartment) of the experimental area that were divided by wire mesh. Hence, actors and recipients were physically separated from each other but in visual contact with each other. Test dyads were physically and visually separated from the rest of the group. The testing apparatus consisted of two boxes (length: 20 cm, width: 5 cm, height: 3 cm) made of acrylic glass with a likewise transparent lid that could be slid open to only one side hereby giving access to the contents of the box. Sliding the lid open was achieved by pulling the end of the lid which was only possible from the actor's compartment. Birds in the recipient compartment never had the possibility to open the box. A transparent partition separated each box into two equally sized fractions which were either baited with a food item or empty, depending on the test condition. Both boxes were placed onto a wooden platform (60*40 cm, attached at a height of 1.4 m) that extended into both experimental compartments and on which birds readily landed. The boxes were positioned at a distance of 40 cm to each other and centered in a size-matched gap in the separating wire mesh. Food rewards were single pieces of dry cat food which is a highly preferred food for jackdaws and was not part of the birds' maintenance diet.

### Training

Prior to testing, the animals were subjected to a training phase to ensure that they were capable of manipulating the apparatus and that they understood the consequences of their choice. Birds were singly trained in the actor's compartment and training consisted of two steps.

**Figure 5:**
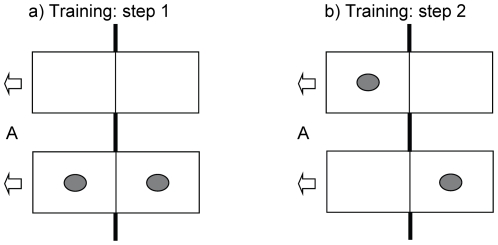
Sketch of baited boxes in both training steps. Grey items display the food reward, A indicates the actor side. Arrows describe the only direction in which the boxes could be opened: only by the actor and to the actor's side. Bold lines above, between, and below the drafted boxes illustrate the dividing wire mesh between test compartments while intermediate thin lines illustrate the transparent partitions within boxes. Distance between the boxes is not to scale. a) represents training step 1, with the 0/0 option on top and the 1/1 option below. b) represents training step 2, with the 1/0 option on top and the 0/1 option below.

In step 1 one box was baited with cat food in each fraction (1/1 option) whereas the other box was left empty (0/0 option, Fig. 5a). Here the individuals learned how to open the box successfully and also that they were unable to reach the food on the recipient's side. Left-right positions of the baited and the unbaited box were counterbalanced throughout the training to avoid the development of a side preference. Birds proceeded to training step 2 after reaching the criterion of 9 correct choices (1/1 option) out of 12 choices on two consecutive days. All birds reached the criterion within two days.

In step 2, one box was baited on the actor's side (1/0 option) and the other box was baited on the recipient's side (0/1 option, Fig. 5b). Here the individuals learned that they were allowed to make only one choice. The experimenter first baited both boxes and then stepped back from the platform, hereby giving way to the bird to land on the platform. As soon as the bird opened one of the boxes by pulling the lid and gained its reward the experimenter approached the platform, causing the subject to leave the platform and hereby learning to make only one choice. A bird proceeded to the test phase after reaching the criterion of 9 correct choices (1/0 option) out of 12 choices on two consecutive days. Birds needed on average 2.3 days to reach the criterion (range: 2–3 days).

### Test

Subjects were tested in 2 conditions. In condition 1, the prosocial condition, one box was baited only on the actor's side (1/0 option) whereas the other box was baited on both the actor's and the recipient's side (1/1 option, Fig. 1a). Opening either of the two boxes would result in delivering food to the actor itself but choosing the 1/1 option would additionally provide food to the recipient and hence, would show prosocial behaviour, as it benefits others but is of little cost to the actor itself [10].

In condition 2, the altruistic condition, one box was baited on the actor's side (1/0 option) and the other box was baited on the recipient's side (0/1 option, Fig. 1b). Here, the actor would gain a food reward only when choosing the 1/0 option but would deliver food only to the recipient when choosing the 0/1 option. The latter choice would be indicative of altruistic behaviour, providing benefits to others while the actor itself does not receive direct benefits but incurs costs [10].

In both conditions subjects were tested in one session with a sibling and in one session with a non-sibling as a recipient, each session consisting of 20 trials. Four subjects were tested first with a sibling (two subjects were tested first in condition 1 and two subjects were tested first in condition 2) and three subjects were tested first with a non-sibling (one subject started with condition 1 and two subjects started with condition 2). A trial was finished when the actor had opened one box and the bird on the respective side had gained and eaten its reward, which was followed by immediate re-baiting by the experimenter and the start of the next trial. If a subject refused to participate in a trial, i.e. did not approach the platform for 3 minutes after baiting, the session was terminated and continued on another day until each subject had finished 20 trials, i.e. had opened one of the boxes 20 times. Left-right positions of the differently baited boxes were counterbalanced throughout the test phase in such a way that the same baiting pattern never occurred more than two times in a row.

### Control

In the control condition subjects were singly tested in the actor's compartment in one session of 20 trials. It resembled the prosocial condition insofar that one box was baited only on the actor's side (1/0 option) whereas the other box was baited on both sides (1/1 option, Fig. 1a) but there was no recipient present. The control condition was interspersed between test conditions and should show whether the birds preferentially chose the box with the greater amount of food even in the absence of a partner.

Subjects opened a box and, depending on the baiting pattern of the chosen box, ate the reward in all test and control trials. Test and control sessions were run in October and November 2007 at which time the birds were sub-adults (5th and 6th month post-fledging) and all trials were videotaped (Sony DCR-TRV14E, Digital Video Camera Recorder).

### Data collection and analysis

We recorded the number of actor's choices for each of the boxes, i.e. how often the actor pulled which box open. Furthermore, we recorded how often the actor was on the platform first and opened one of the boxes prior to the recipient landing on the platform in its compartment (actor-first). We also recorded how often the recipient landed on the platform first which was always followed by immediately positioning itself next to the box baited on its side and/or trying to get access to the food reward by pecking or stepping onto the box and occurred prior to the actor opening one of the boxes (recipient-first).

We analyzed the data by conducting a generalized linear mixed model (GLMM), applying the restricted maximum likelihood procedure (REML). We constructed the GLMM with actor choice (selfish or cooperative: selfish = 1/0 option in both conditions, cooperative = 1/1 option in the prosocial condition, 0/1 option in the altruistic condition) as the binomial response variable. Selfish choices were coded as 0, cooperative choices were coded as 1. Into the model we entered subject identity as random term to account for repeated measures. We included “first approach”, “condition”, “kinship”, “sex relation of dyad”, and “sex of actor” as fixed terms and combinations thereof as first order interactions. We further included “first approach*condition*kinship”, “first approach*condition*sex relation of dyads” and “first approach*condition*sex of actor” as second order interactions to explicitly test for our hypotheses. We sequentially deleted fixed terms in order of decreasing significance, starting with second order interaction terms in the model. Only terms with *P*<0.05 or *P*<0.1 (to account for tendencies) and, if applicable, corresponding lower order interactions and main terms were kept in the model. Excluded terms were reentered one by one into the final model to confirm that these terms did not explain a significant part of the variation.

To compare selfish versus cooperative choices in actor- and recipient-first trials and in the control condition we used Wilcoxon signed-ranks tests with α<0.05. All analyses were performed using SPSS 19 (IBM SPSS Statistics 19.0.0).
